# Seasonal heterogeneity of ocean warming: a mortality sink for ectotherm colonizers

**DOI:** 10.1038/srep23983

**Published:** 2016-04-05

**Authors:** Fulvio Maffucci, Raffaele Corrado, Luigi Palatella, Marco Borra, Salvatore Marullo, Sandra Hochscheid, Guglielmo Lacorata, Daniele Iudicone

**Affiliations:** 1 Stazione Zoologica Anton Dohrn, Villa Comunale, 80121, Naples, Italy; 2 Dipartimento di Scienze, Università Roma Tre, Viale G. Marconi 446, 00146 Rome, Italy; 3 Consiglio Nazionale delle Ricerche, Istituto di Scienze dell'Atmosfera e del Clima, Str. Lecce-Monteroni, 73100, Lecce, Italy

## Abstract

Distribution shifts are a common adaptive response of marine ectotherms to climate change but the pace of redistribution depends on species-specific traits that may promote or hamper expansion to northern habitats. Here we show that recently, the loggerhead turtle (*Caretta caretta*) has begun to nest steadily beyond the northern edge of the species’ range in the Mediterranean basin. This range expansion is associated with a significant warming of spring and summer sea surface temperature (SST) that offers a wider thermal window suitable for nesting. However, we found that post-hatchlings departing from this location experience low winter SST that may affect their survival and thus hamper the stabilization of the site by self-recruitment. The inspection of the Intergovernmental Panel on Climate Change model projections and observational data on SST trends shows that, despite the annual warming for this century, winter SST show little or no trends. Therefore, thermal constraints during the early developmental phase may limit the chance of population growth at this location also in the near future, despite increasingly favourable conditions at the nesting sites. Quantifying and understanding the interplay between dispersal and environmental changes at all life stages is critical for predicting ectotherm range expansion with climate warming.

Anthropogenic induced climate warming is projected to be a major challenge to Earth’s biota in the 21^st^ century^1^. Despite slower ocean warming over the last 50 years, the average speed of isotherm migrations at the ocean surface has been as fast as or even faster than the terrestrial counterpart^2^. Shifts in species’ distributions have been observed in many different marine taxa and are considered as one of the key adaptive mechanisms to endure changes in ambient temperature^3^,^4^. In particular, marine ectotherms, being thermal range conformers, are predicted to expand poleward with ongoing warming as new locations that were previously too cold for survival will become suitable for colonists^5^. However, the pace of redistribution depends on the cumulative effects of climate warming on all ontogenetic life stages that may occupy different habitats, differ in behavioural traits and/or thermal sensitivity^6^.

In this respect, the endangered loggerhead turtle is an interesting example^7^. This species possesses the widest nesting range among marine reptiles spanning from tropical to temperate latitudes. As all extant sea turtle species, the loggerhead turtle has survived major climate changes in the past by altering the equilibrium between colonization and local extinction, but how and whether it is responding to present day climate warming is still under debate^8^,^9^,^10^. Loggerhead turtles, as well as salmon and many other marine and terrestrial animals, exhibit philopatry, i.e. they return to their natal sites, a strategy that increases reproductive success but sets certain limits to genetic diversity and adaptability of populations^11^,^12^. Philopatric animals rely on imprinting mechanisms during the early life stage to recognize site specific environmental features of their birth area that guide them home. Poleward expansion of the nesting range must therefore occur through self-recruitment, which means that the occasional nesting of straying turtles at higher latitudes is associated with the production of female offspring that survive to nest decades later at the same location^12^,^13^,^14^. Given the influence of sand temperatures on embryonic development, it is not surprising that the majority of studies on climate induced range shifts have focused on the nesting beaches^10^,^15^,^16^. However, other factors might create a barrier towards the colonization of northern habitats. During the first year of their life, sea turtles possess limited swimming capacities and mostly rely on ocean currents to disperse towards suitable developmental habitats^17^. Therefore, if thermal conditions encountered along their dispersal pathways are not favourable for post-hatchling survival, nesting range expansion may be impeded, especially at more poleward sites.

Located at the northern edge of the species’ range, the Mediterranean population is the result of at least two independent immigration events from Atlantic rookeries and it has endured climatic oscillations since the Pleistocene by shifting its nesting range in accordance with the migration of its thermal niche^18^. Until now regular nesting has occurred exclusively in the warmer eastern basin, mainly in Libya, Greece, Turkey and Cyprus^19^. However, in the last two decades, an increasing number of sporadic nests have been documented in the Western Mediterranean in the proximity of foraging habitats along the Spanish or Italian coasts (Fig. 1 and Supplementary Table S1). Sporadic nests are usually scattered in time and space and may represent a mechanism by which the species explores new locations and expands the nesting range if environmental conditions have become conducive for embryonic development^14^.

Here we concentrate on the Campanian coast (SW Italy), an important foraging habitat for loggerhead turtles of Atlantic and Mediterranean origin^20^ and the only region in the Western Mediterranean where nesting is now being recorded each year. The first nest was discovered in 2002 and since then single nests were found every 2–4 years. However, since 2012 nesting became more regular with 1–7 nests laid each year (N = 16), all of which were located along a 60 km strip of coastline at the southern portion of the study area (Fig. 1). Mean hatching success of the first discovered nests (N = 4) was 62.6% and increased to 85.1% during the last three years (N = 8, excluding one inundated nest, see Table 1). We also found that, for clutches laid in the same week of July, incubation durations decreased by 8–16 days compared to nests before 2013 (Table 1). This coincided with generally higher incubation temperatures during the most recent years. In fact, mean temperatures of the middle third of incubation ranged between 28.4 and 31.1 °C, and were thus within the optimal range of hatchling production as determined in controlled laboratory experiments^21^. Considering the pivotal incubation temperatures, 28.9 °C^22^ and 29.3 °C^23^, and durations, 59.9^22^ and 56.6^23^ days (i.e. the values producing 50% of each sex) for the Mediterranean populations, it appears that conditions have been conducive to female production with some nests approaching even a balanced sex ratio (Table 1). Taken together, these results support the predictions of good habitat suitability for nesting in the study area^24^ where incubation conditions have improved substantially during the monitoring period even though the low number of nests precludes any robust statistical analysis. The mean straight carapace length (SCL = 40.5 ± 3 mm, range 28–49 mm, N = 682) and mean body mass (M_b_ = 15.1 ± 1.1 g, range 11.2–20 g, N = 569) of the hatchlings from eleven Campanian nests were comparable to those recorded at eastern Mediterranean nesting beaches^25^,^26^,^27^, while hatchlings were smaller and lighter than those from Atlantic rookeries^28^. This may suggest that the females were indeed of Mediterranean origin, which is also consistent with previous results of mixed stock analysis of loggerhead turtles foraging along these coasts showing that the vast majority of the individuals belongs to local rookeries^29^. However, the contribution of Atlantic females could not be definitively excluded based on results of the genetic analysis. In fact, samples from 12 out of 13 nests exhibited mtDNA haplotypes that are widely shared between Mediterranean and Atlantic rookeries (CC-A2.1 and CC-A3.1, Table 1)^30^. Moreover, although the other haplotype found at this site (CC-A10.4) has been reported exclusively from Atlantic nesting beaches^30^, its shorter sequence (CC-A10) has been found at low frequency in Greece^31^ which suggests its probable presence also in the Mediterranean Sea. Because of the opportunistic sampling of genetic materials from the sporadic nests, pseudo-replication (i.e. the repeated sampling of the same female during a single nesting season or in different years) may be an issue. However, using photo identification it was possible to ascertain that at least six different females nested over the last eight years (Supplementary Fig. S1), four of which were observed only in 2015. Therefore, the occurrence of several nesting females in the study area indicates multiple explorations outside the regular nesting areas.

The SST trend from 1854 to 2014 shows that the south Tyrrhenian Sea has steadily warmed up after the last minimum in 1978 whereby the last three years were amongst the warmest on record (Fig. 2). Contemporaneously the number of nests in the study area have increased, particularly during the last three years, a phenomenon that could be associated to the changes in SST. Indeed, this parameter strongly influences loggerhead nesting phenology and sets the width of the optimal thermal window for nesting^32^. In particular, the months between April and July exhibited the highest rates of warming with values ranging from 0.035 °C/year to 0.050 °C/year (Fig. 3a). Although water temperature is not the exclusive factor that triggers the start of the reproductive season, it is known that higher SSTs during spring provoke earlier nesting^33^. Thus, mature adult turtles foraging at these latitudes could encounter appropriate conditions for mating and nesting opportunistically^32^ already at the beginning of the summer season. This is supported by a recent observation of two mating loggerhead turtles in the Gulf of Naples during May 2015, which is the first time that this behaviour has been reported in the Western Mediterranean. Moreover, the combination of rapid warming in spring and only moderate warming in summer results in a wider temporal window of suitable thermal conditions that may benefit embryonic development and female offspring production. This implies that individual loggerhead turtles possess sufficient plasticity in their phylopatric behaviour to exploit nesting beaches that have become suitable with climate warming^34^. In conclusion, given that the conditions for nesting are already good and considering that they are predicted to further improve in our study area^24^, we may indeed be witnessing a colonisation event that is initiating right now.

However, the increased frequency of occasional nesting due to climate warming is not sufficient to stabilize the nesting site in the future if self-recruitment rates are very low^35^. Survival of post-hatchlings during the surface pelagic phase is necessary to sustain nesting range expansion. Using numerical simulation of post-hatchling dispersal, we found a wide spreading with most of the individuals accumulating in the south Tyrrhenian Sea during the first year of their life. The drifting pattern was consistent among all eight simulation years. In addition, we found two previously undescribed southward conveyors connecting the south Tyrrhenian Sea with the Strait of Sicily and thus with the favourable Eastern Mediterranean developmental habitat (Fig. 4). These transport mechanisms cannot be inferred from the mere knowledge of the mean (climatological) current patterns since they are the result of nonlinear dynamic processes that are potentially relevant for the inter-basin connectivity^36^.

We then estimated hatchling survival along drifting trajectories using a published mortality function based on SST, which resulted in a mean survival index of 4.4% (Supplementary Fig. S2). This is because only those individuals that crossed to the Eastern Mediterranean through the southward channels survived in all simulation years, which highlights the unsuitability of the Western Mediterranean as a nursery area. Indeed, survival indices in the south Tyrrhenian Sea were about 17 times lower than those obtained for hatchlings departing from Libya (mean SI = 68%), and 7 times lower than SI of hatchlings from Greece (mean SI = 26%). The latter had lower SI because substantial, albeit variable proportions of hatchlings enter the nearby Adriatic Sea and reach the northern part, which is also characteristic for cold winter temperatures. Directional swimming, even by small turtles, can impact their oceanic movements^37^,^38^ and may lead to different hatchling distribution patterns than the ones we modelled and, possibly, to higher SI’s. However, directional swimming by hatchlings is not described well enough for the populations considered here to accurately parametrize in our model. This issue remains important to resolve and future work should focus on developing the dispersal simulations to include a realistic representation of turtle behaviour^39^. Despite significant improvement of the conditions during the breeding period in the last decades, winter is currently the key determinant of species range expansion with climate warming because it drastically reduces the number of individuals that may potentially self-recruit to this nesting location in the future.

We then addressed the question of the future of the nursery area suitability in a climate change context, while duly considering the importance of the winter season. First, by rerunning the simulations adding a bias to the winter temperatures, a temperature increment of 1.5 °C to 2.25 °C during the colder months is required to obtain an SI comparable to the 26% and 68% benchmark values obtained for Greece and Libya, respectively (Supplementary Fig. S2). Considering the future estimates provided by the Coupled Model Intercomparison Project 5 (CMIP5)^40^,^41^, RCP 4.5, for the SST in the south Tyrrhenian Sea, the 15 °C degrees threshold in monthly SST minima is expected to be reached relatively early in this century (median value: 2030). However, as a whole the historical CMIP5 simulations show similar warming trends for winter and spring temperatures, in contrast with the observed seasonal trends (Fig. 3a). Since the CMIP5 monthly trends for the region have a phase similar to the Atlantic Multidecadal Oscillation (AMO) intensity (i.e., the SST anomaly over the North Atlantic; Fig. 3a), the difference to the observations is possibly due to limitations in the Mediterranean basin physics in CMIP5, which basically projects the Atlantic tendencies into the Mediterranean. This tendency is maintained also in the CMIP5 forecasts for the 21^st^ century^42^. On the contrary, the observed winter SSTs do not show a significant increase during the last decades and a statistical forecast of winter SST does not highlight any positive trend for the future (Fig. 3b, Supplementary Fig. S3). Given the mismatch between the CMIP5 description of the recent state of the system and the observational data, we conclude that it is not possible to forecast when the thermal conditions at this post-hatchling developmental habitat will become sustainable. While we acknowledge that most of this difference is known and well explained^43^, here we emphasise the need to further understand the limitations to the capability of capturing the seasonal response for regional seas. This is particularly important because these simulations are used to force regional models which are then used to predict local impacts of climate change.

Our study showed data in support of the existence of a Mediterranean hot spot for poleward expansion of loggerhead turtles. Through the multi-disciplinary study on the past, present and future thermal conditions in the potential expansion area, we found that inspecting the climate impact on the nesting sites is not enough to evaluate the potential for stable expansion. As demonstrated here, a complete assessment should consider the interplay between the physiological capacities of a species, like temperature sensitivity, and the environmental conditions of all major habitats utilized by the species, especially during the first life stage.

Furthermore, the winter season as a key factor has been overlooked in the past. The physics of the seasonal response to climate change per se are still unclear. Indeed, the marked seasonality we found is coherent with the large mixed layer differences (thermal inertia) between winter and spring in the region. The different dynamics of winter and spring SST responses to climate change may lead to a conundrum where the number of exploratory females and hatching success will increase with climate warming but the stabilization of this northern nesting site will still be hampered by high mortality rates of post-hatchlings due to the persistence of low winter temperatures. Clearly, this seasonal response to climate change might have implications for other marine organisms with a complex life cycle and further investigations on the occurrence of seasonally dependent trends should be conducted in other basins as well as on other marine organisms.

## Methods

### Nest sites, incubation temperatures and hatchling measures

The Sea Turtle Stranding Network in the Campania Region (SW Italy) has been operating since 1983, including the collection of all data on loggerhead turtle nests in the study area (41.22°N, 13.76°E–40.04°N, 15.64°E). These were reported by private citizens who observed either the female during nesting or the hatchlings during their emergence from the nest. Incubation temperatures were monitored at 1-h intervals in the former nests by placing data storage tags (Cefas Technology Ltd, Lowestoft, UK; and i-Button DS1923-F5, Maxim Integrated Products, Dallas Semiconductor, Sunnyvale, CA) directly at the top of the egg chamber (Table 1). After emergence from the nest hatchlings were weighed to the nearest 0.1 g with a digital balance (model GF-300, A&D Engineering, San Jose, CA, 0.01 g accuracy). Straight carapace length (SCL) was measured using callipers (0.1 mm accuracy).

### MtDNA analysis

Tissue samples for genetic analysis were obtained from 13 out of 18 nests laid since 2002. Genomic DNA was extracted using NucleoSpin Tissue kit (Macherey-Nagel, Duren, Germany) following the manufacturers’ protocols and purified by binding and eluting to a silica membrane using vacuum filtration. A 850 bp fragment of the mtDNA, encompassing tRNAThr, tRNAPro and the control region, was amplified and sequenced following Saied *et al.*^44^. The resulting sequences were compared to the online haplotype registry that is maintained by the Archie Carr Center for Sea Turtle Research of the University of Florida (ACCSTR; http://accstr.ufl.edu/ccmtdna.html). The mtDNA haplotype of the 2002 nest was determined by Carreras *et al.*^34^.

### Photo identification of nesting females

As stated above, all except one (the female that nested on 29 July 2015 in Marina di Camerota, see Table 1) nesting turtles were observed by private citizens who never reported the presence of flipper tags which could aid to identify the turtles. However, they took photos or made videos of the nesting turtles (N = 9). As with other sea turtle species, loggerhead turtles have a cluster of scales on the dorsal and lateral surfaces of the head that form unique scale patterns. Photos (n = 4) or still frames extracted from videos (n = 3) of the nesting females’ left side of the head, which was the most available view, were aligned to compare individual scale patterns using Adobe Photoshop© CS6 imaging software (version 13.0 × 64, Adobe Systems Inc.). Two out of nine turtles had to be excluded from this analysis because no images of the left side were available. The images were visibly assessed for clear marks of differences to identify different turtles (Supplementary Fig. S1).

### Lagrangian simulations

Numerical simulations of Lagrangian transport in the marine upper layer have been performed using the Mediterranean Forecasting System (MFS) analysis fields (velocities, temperature) for the years from 2006 to 2013. The MFS provides the basin-scale circulation field at 6.5 × 6.5 km spatial, and 1 day temporal resolution. Mesoscale two-dimensional turbulent dispersion has been parameterized with an established state-of-the-art kinematic Lagrangian model (KLM), suitable to simulate any kind of two-particle dispersion regime. In our case, the KLM has been calibrated on Mediterranean drifter trajectory data as in Lacorata *et al.*^45^. Another 3D KLM is added in order to simulate the small scale three dimensional turbulence on the scale of the mixed layer as in Palatella *et al.*^36^. The initial conditions of 25600 trajectories have been distributed off-shore (between 10 and 50 km from the coast) along a 100 km line in the vicinity of the study area, at 3 m depth. The starting dispersion is intended to simulate the position of the turtle hatchlings after the first one/two days of swimming activity. After this period the hatchlings are considered as passive buoyant tracers that follow constant depth currents, at least for the first year of life. A mortality rate is defined depending on the sea surface temperature along the Lagrangian trajectory: turtles experiencing mean SST < 15 °C for more than 10 days had a 50% chance of survival for each extra day at the same conditions while those individuals that encountered SST < 10 °C died instantly^46^. The fraction of hatchlings that survived after a year of transport is defined as the survival index (SI). The SI, and so the stability of the release site, depends ultimately on the interaction between Lagrangian transport and SST fields, at given climate conditions. To compare these results to hatchling dispersions from regular nesting sites in the Eastern Mediterranean we repeated these simulations for two further sites, one in Zakynthos, Greece, which hosts the largest loggerhead rookery in the Mediterranean, and one in Sirte, Libya, which hosts one of the founder rookeries for the Mediterranean nesting population^18^,^19^.

### Sea surface temperature analysis

Sea surface temperature time series of the south Tyrrhenian Sea have been obtained from three different sources spanning over different time windows and with different spatial resolutions: Extended Reconstructed Sea Surface Temperature (ERSST) from the NOAA National Climatic Data Center (1854 to the present), Hadley Centre Sea Ice and Sea Surface Temperature data set (HadISST) from Met office Hadley Centre (1870 to the present) and satellite reconstructed (interpolated through an Optimal Interpolation algorithm) AVHRR Pathfinder daily SST data from MyOCEAN-Copernicus (from 1982 to 2012). The three time series follow quite closely each other. They basically describe the same history both in terms of trends and of multi-decadal oscillations and can consequently be used interchangeably to study the long-term variation of the Tyrrhenian Sea SST field. For this reason, rather than choosing one of the three time series, we decided to adjust both monthly ERSST and HadISST using a simple slope and bias to monthly pathfinder SSTs and then we averaged the three time series to produce a single monthly time series running from January 1854 to December 2014.

**Figure 2:**
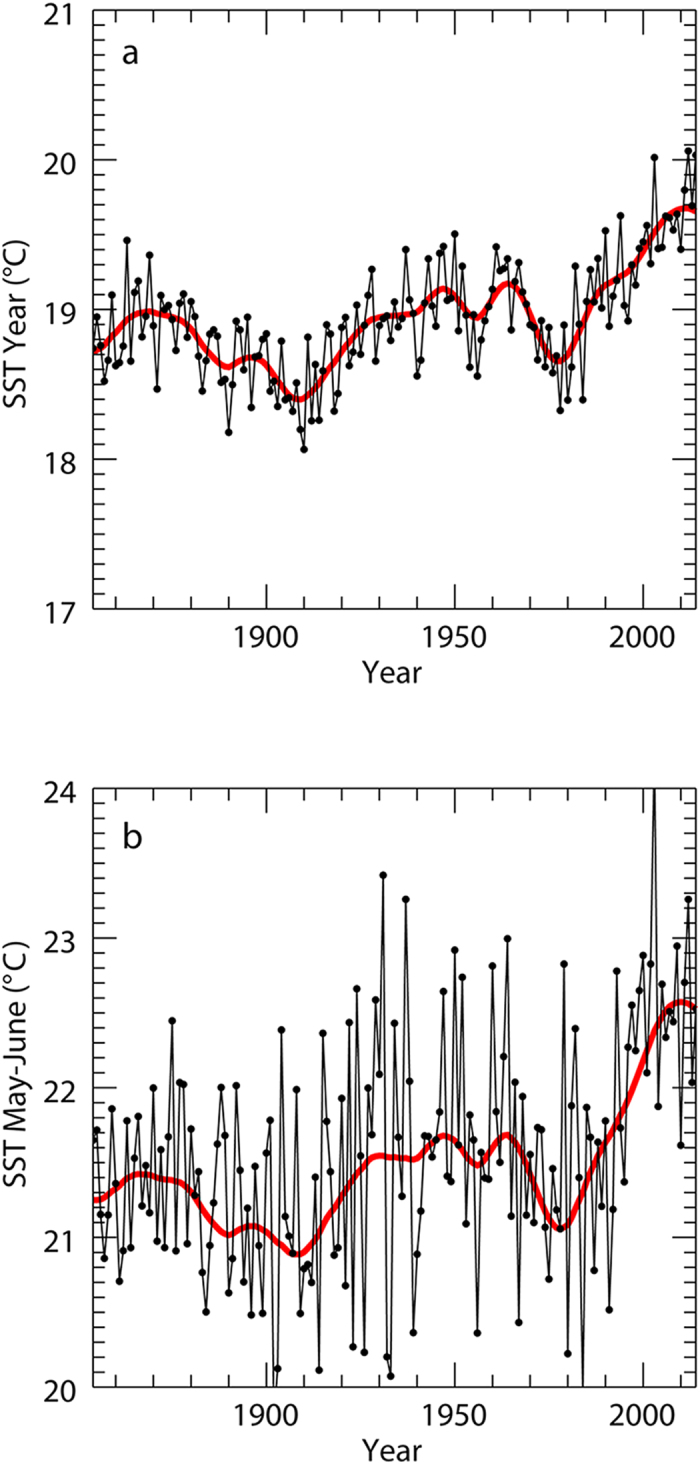
Sea surface Temperature (SST) trends in the south Tyrrhenian Sea from 1854–2014. Annual (**a**) and spring (**b**) (May–June) SST values. Singular Spectral Analysis combined with Maximum Entropy Method was used to estimate the oscillatory components in the time series.

Singular Spectral Analysis (SSA) was used to separate trends and oscillatory modes (red curve in Fig. 2a,b) from noise in the SST time series.

### CMIP5 data usage

From 34 Atmosphere-Ocean General Circulation Model (AOGCM) and ESM model's output, all prepared for CMIP5, RCP 4.5 (see Supplementary Table S2), we extract monthly mean SSTs for South Tyrrhenian Sea (12° < Longitude < 16° E, 38° < Latitude < 41° N) since January 1861 to December 2100. Data were in form of time series, then we determined, for each model's output, when monthly SST minimum exceeds the 15 °C degrees threshold for post-hatchling survival (see Lagrangian simulations). Since we were interested in SST trends only, our analysis does not consider any bias correction.

## Additional Information

**How to cite this article**: Maffucci, F. *et al.* Seasonal heterogeneity of ocean warming: a mortality sink for ectotherm colonizers. *Sci. Rep.* **6**, 23983; doi: 10.1038/srep23983 (2016).

## Supplementary Material

Supplementary Information

Supplementary Figure S4a

Supplementary Figure S4b

Supplementary Figure S4c

## Figures and Tables

**Figure 1:**
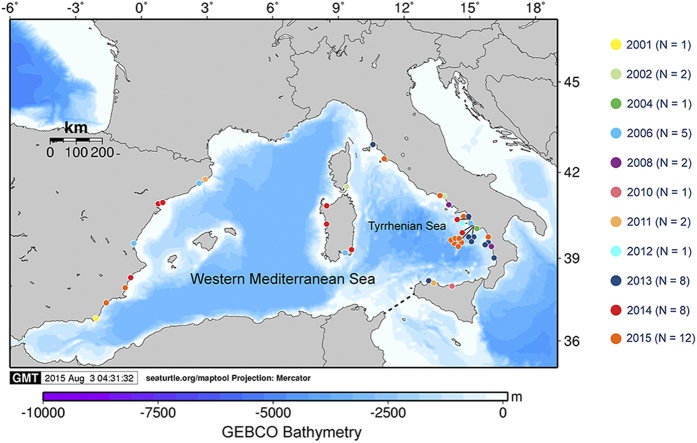
Occasional nesting by loggerhead turtles in the western Mediterranean since 2001. Only confirmed nests for which either nest chamber or egg remains were found are reported. Each dot represents a single nest with the different color indicating the year of deposition. Our work focussed on the South Tyrrhenian coasts, the only area where nesting regularly occurrs since 2012. For more information see Table 1 and Supplementary Table S1. This map was created using the free Maptool program available at www.seaturtle.org.

**Figure 3:**
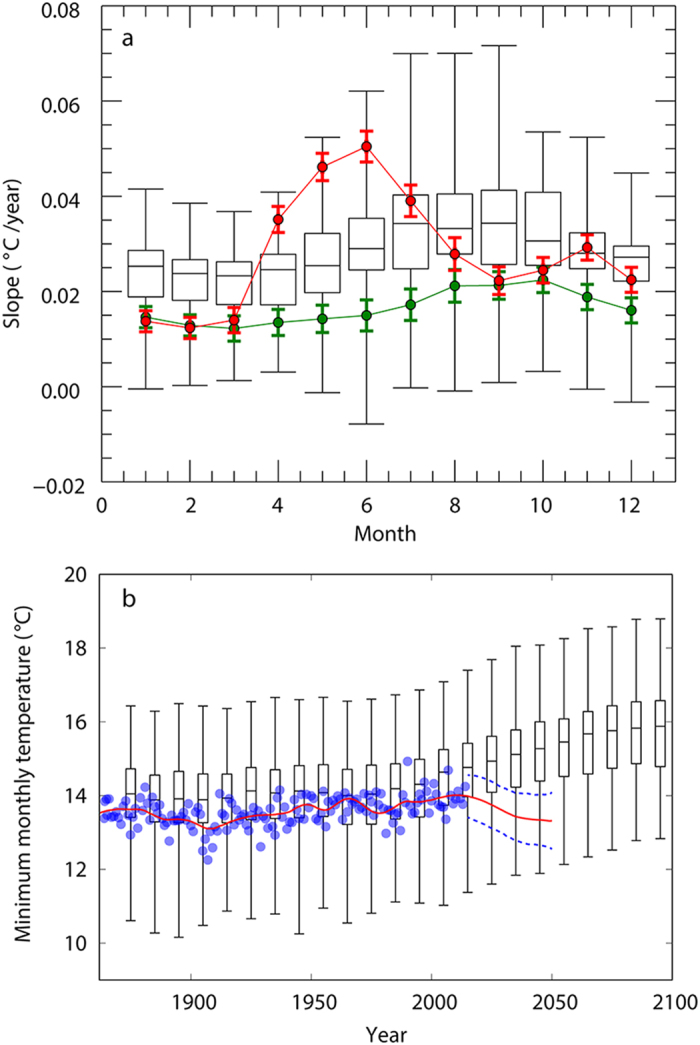
Seasonal variability of the SST trends. Monthly SST trends during the 1982–2014 period (**a**). Box plots represent the graphical summary obtained from all CMIP5 simulations. In red: trends in observed SSTs in the study area; in green: monthly trends for the AMO (without detrending). The latter is representative of the observed SST in the Atlantic north of the equator. (**b**) Minimum monthly temperature calculated from measured and reconstructed SST time series (blue circles) with superimposed Singular Spectral Analysis reconstructed signal plus the statistical prediction (red line) with the corresponding error (blue dotted lines). The black box plot refers to the minimum monthly temperature calculated from the different GCM of the CMIP5 ensemble. They indicate the minimum, the 75% percentile, the median, the 25% percentile and the maximum value inside the model ensemble.

**Figure 4:**
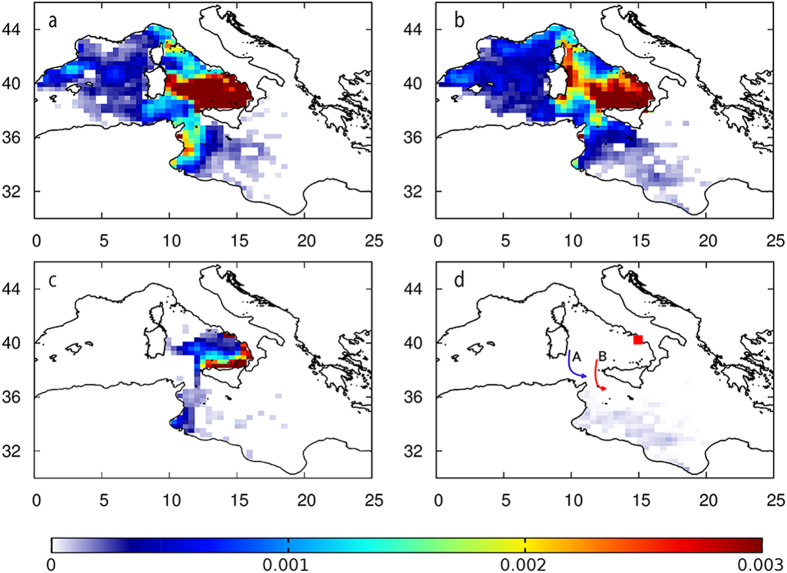
Modelled dispersion of hatchlings departing from the study area. Relative density (%) after one year of dispersal in 2007 (**a,c**) and in 2013 (**b,d**), obtained either without (**a,b**) and with (**c,d**) the mortality function based on SST (for animated dispersion see Supplementary Fig. S4). The winter (blue) and summer (red) southward conveyors connecting the south Tyrrhenian Sea with the Eastern Mediterranean are shown in Panel d. Cumulative curves of final latitude and longitude are presented in Supplementary Fig. S5. The maps were created using the free online software Gnuplot version 4.6 (Copyright 1986–1993, 1998, 2004 Thomas Williams, Colin Kelley, http://www.gnuplot.info/) and assembled with Gimp (http://www.gimp.org/).

**Table 1:** Summary data of loggerhead turtle nests in the Campania Region, SW Italy, between 2002 and 2015.

Site	Date of egg laying	Date of 1^st^ emergence	Incubation duration	Clutch size	Mean SCL ± s.d. (cm)	Mean M_b_ ± s.d. (g)	Emergence success	Hatching success	Mean middle third T (°C)	Note	mtDNA haplotype
Baia Domizia	11/07/2002	–	64	92	–	-	–	49.4	27.4	Relocated	CC-A10.4
Marina di Camerota	–	17/10/2004	–	–	–	–	–	–	–	Egg remains	–
Ogliastro	25/07/2006	–	73	93	4.1 ± 0.1	14.7 ± 1	28.0	33.3	27.1	Relocated	–
Lucrino^a^	15/07/2008	–	46	115	4 ± 1.2	15.2 ± 0.5	88.7	92.2	34.5	Incubated on a geothermal beach	–
Ogliastro	–	28/08/2012	–	73	4 ± 0.7	15.5 ± 1.9	61.6	75.3	–		–
Palinuro	–	19/08/2013	–	132	4 ± 0.1	14.9 ± 0.7	40.9	62.1	–	Escavated	CC-A2.1
Palinuro^b^	15/07/2013	–	56	96	3.9 ± 0.1	14 ± 0.7	99.0	99.0	28.9		CC-A2.1
Battipaglia	–	12/10/2013	–	110	–	–	–	–	–	Escavated	CC-A2.1
Palinuro	–	06/12/2013*	–	48^#^	–	–	–	–	–	Predated	CC-A2.1
Acciaroli^b^	30/07/2014	–	60	118	4.3 ± 0.4	–	94.9	95.8	28.4		–
Capaccio	–	25/08/2014	–	117	4.1 ± 0.1	14.8 ± 0.7	88.9	91.5	–		CC-A2.1
Marina di Camerota^c^	19/06/2015	–	50	99	3.9 ± 0.3	15.3 ± 0.6	83.0	83.0	31.1		CC-A2.1
Marina di Camerota	07/07/2015	–	58	60	–	–	80.0	80.0	–		CC-A2.1
Ascea Marina^d^	18/07/2015	–	56	87	3.9 ± 0.3	14.6 ± 0.5	80.5	80.5	29.0	Relocated	CC-A3.1
Eboli	–	28/08/2015	–	–	–	–	–	–	–	Predated	CC-A2.1
Marina di Camerota^e^	29/07/2015	–	58	56	4.1 ± 0.1	16.1 ± 0.1	87.5	89.3	29.9		CC-A2.1
Ascea Marina^f^	29/07/2015	–	57	55	4 ± 0.1	16.2 ± 0.5	78.2	78.2	29.1		CC-A2.1
Ascea Marina	30/07/2015	–	62	58	3.8 ± 0.1	13.2 ± 0.8	15.5	25.9	29.2	Inundated, relocated	CC-A2.1

Either the date of egg laying or first hatchling emergence are given, depending on which occasion the nest was detected. Incubation duration is the time that elapsed between the date of egg laying and the first hatchling emergence. Also given are straight carapace length (SCL) and body mass (M_b_) of hatchlings, mean incubation temperature (T) during the middle third of the incubation period and mtDNA haplotypes for an 800 bp sequence (see Methods for further details on how measures and samples were collected).

^#^Estimated from remains of predated nest.

*Date nest was discovered, hatching date is unknown.

^a-d^Different superscript letters indicate different individuals, same letters indicate same turtle, as determined through photo identification (see Supplementary Fig. S1).
