# Supplementary material for: Seasonal heterogeneity of ocean warming: a mortality sink for ectotherm colonizers
Source: Sci Rep. 2016 Apr 5;6:23983. doi: 10.1038/srep23983 (PMC4820753; doi:10.1038/srep23983)
Supplement: Supplementary Information [file srep23983-s1.pdf]

Supplementary information

Seasonal heterogeneity of ocean warming: a mortality sink for ectotherm colonizers

Authors:

Fulvio Maffucci[1,2], Raffaele Corrado[3], Luigi Palatella[3], Marco Borra[1], Salvatore Marullo[2], Sandra Hochscheid*[1], Guglielmo Lacorata[3]*, Daniele Iudicone*[1]

*corresponding authors E-mail addresses: sandra.hochscheid@szn.it; guglielmo.lacorata@gmail.com; daniele.iudicone@szn.it

Affiliations

1 Stazione Zoologica Anton Dohrn, Villa Comunale, 80121, Naples

2 Dipartimento di Scienze, Università Roma Tre, Viale G. Marconi 446, 00146 Rome

3 Consiglio Nazionale delle Ricerche, Istituto di Scienze dell'Atmosfera e del Clima, Str. Lecce-Monteroni, 73100, Lecce, Italy.

4 Agenzia nazionale per le nuove tecnologie, l'energia e lo sviluppo economico sostenibile, ENEA — Centro Ricerche Frascati, Frascati, Italy

Supplementary Figures

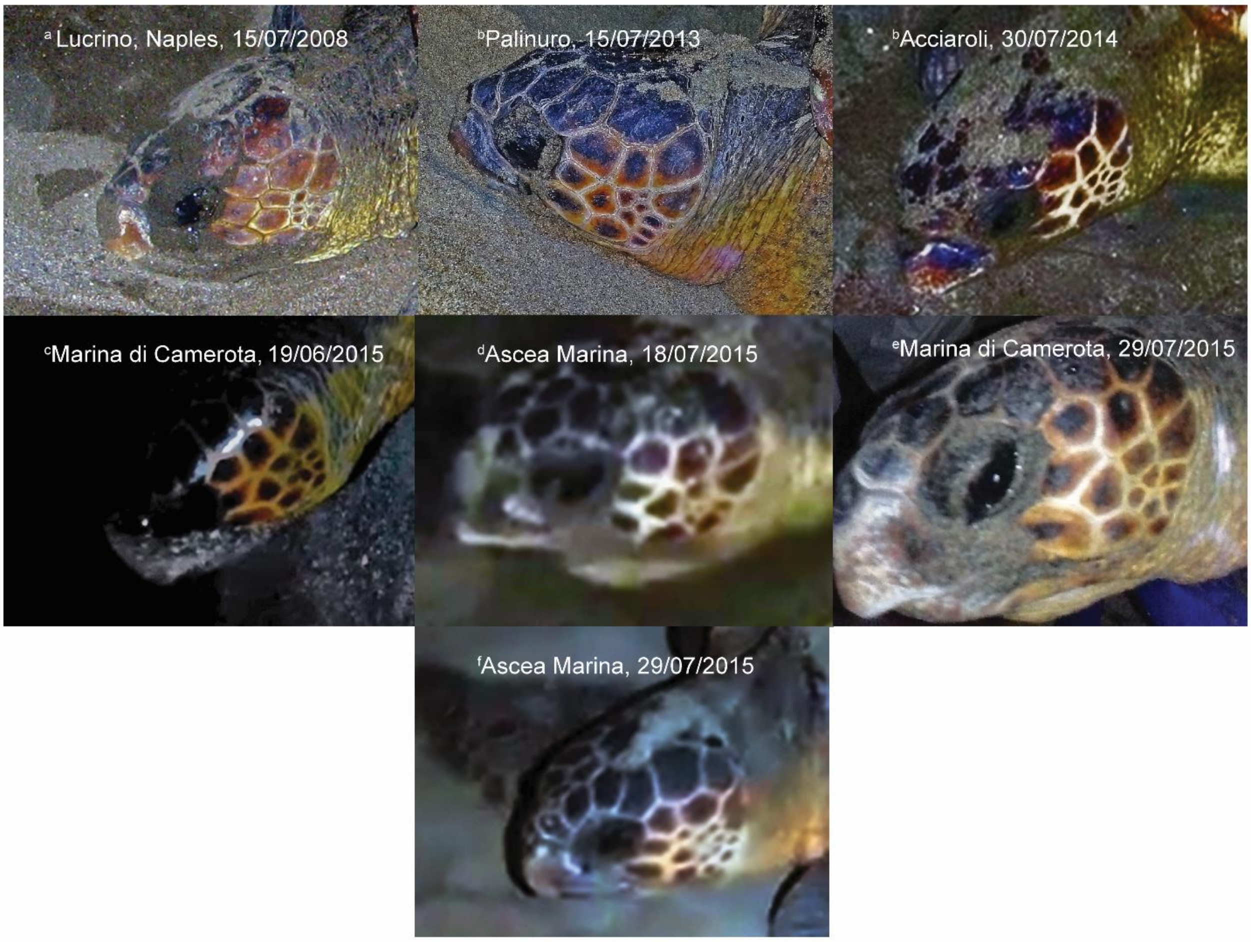

Supplementary Figure S1. Photo id of occasional nesters. Lateral view of left head scales are shown in comparison to distinguish between individual females. * indicates possible match. Photo quality varies according to original material available.

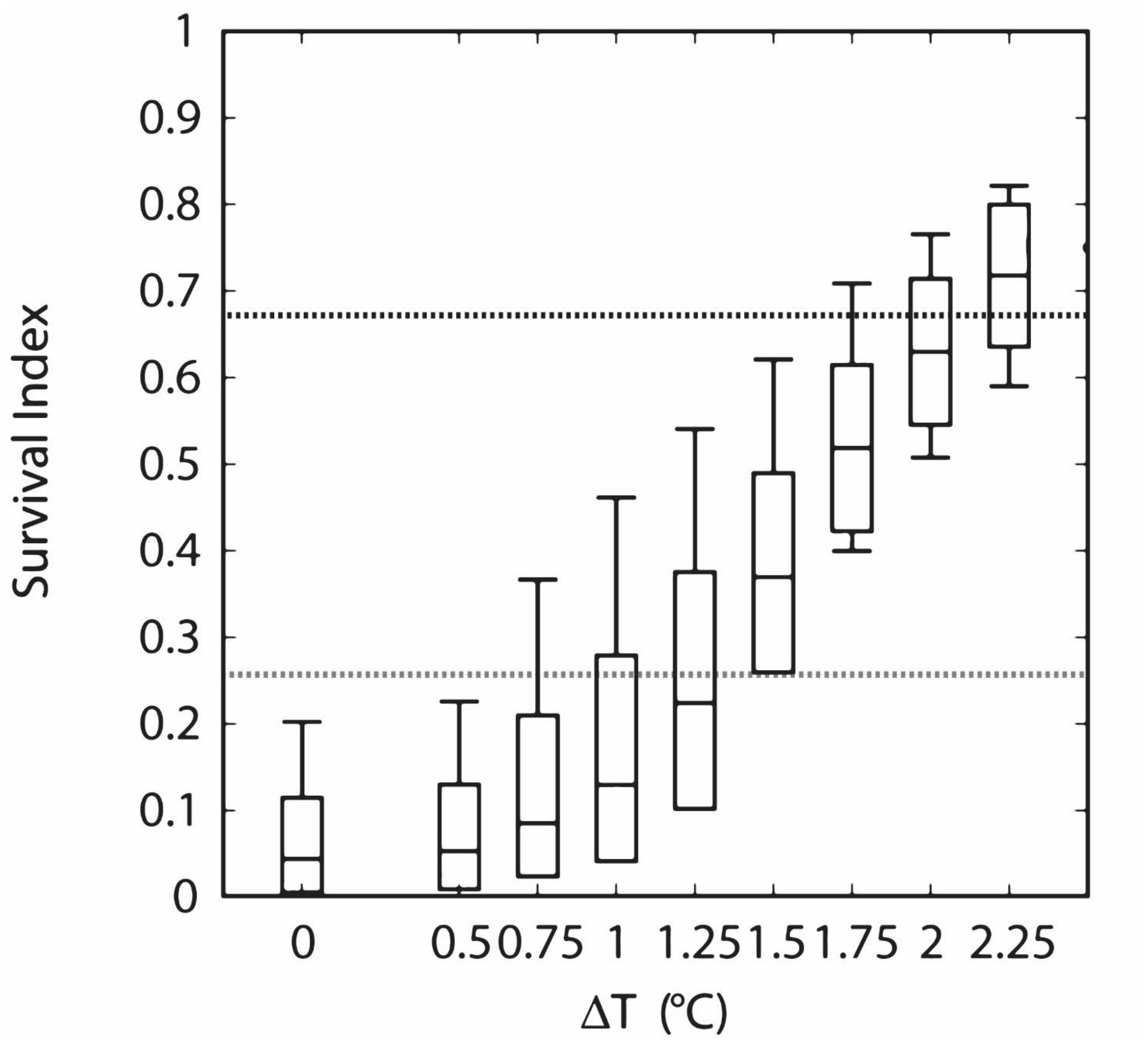

Supplementary Figure S2. Box plot of the Survival Index distribution (SI) for the period 2006-2014 at systematic SST increments. Each box represents: minimum, mean-standard deviation, mean, mean+standard deviation, maximum. The black and grey horizontal lines are the equivalent SIs for Libya and Greece, respectively.

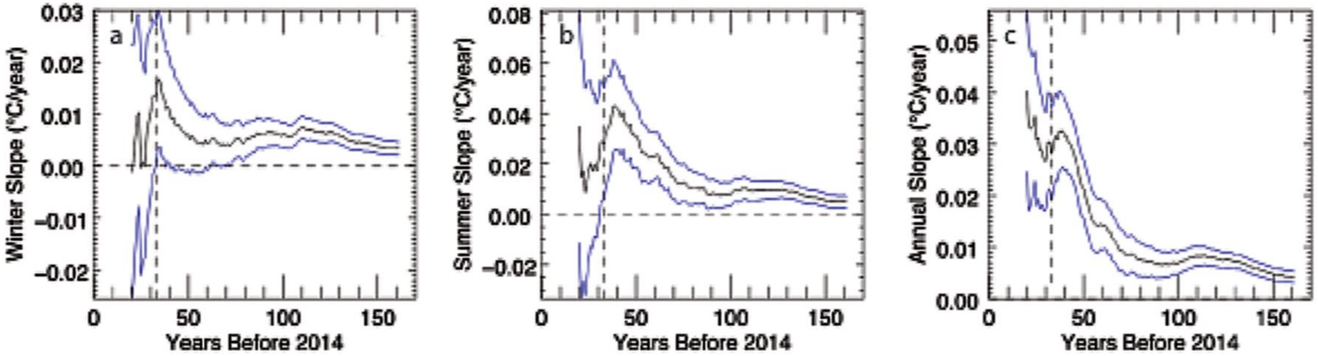

Supplementary Figure S3. Linear trend estimates of the South Tyrrhenian Sea SST time series as a function of the number of years before 2014 used for the fit. (a) winter minima, (b) summer maxima, (c) annual average. The blue lines delimitate the 95% confidence limit of the slope of the linear fit. The dashed line indicates the year when remote sensing through satellites began.

Supplementary Figure S4. Animations showing the Lagrangian simulations for 2006-2007 (a), 2008-2009 (b) and 2010-2011 (c). The color shows the SST experienced by each particle along its path. The maps were created using the free online software Gnuplot version 4.6 (Copyright 1986 - 1993, 1998, 2004 Thomas Williams, Colin Kelley, http://www.gnuplot.info/)

Note: This figure is contained in three *.gif files (supplementary figure S4a, supplementary figure S4b, supplementary figure S4c). The files were uploaded separately as videos since they contain animations which do not show in the pdf format.

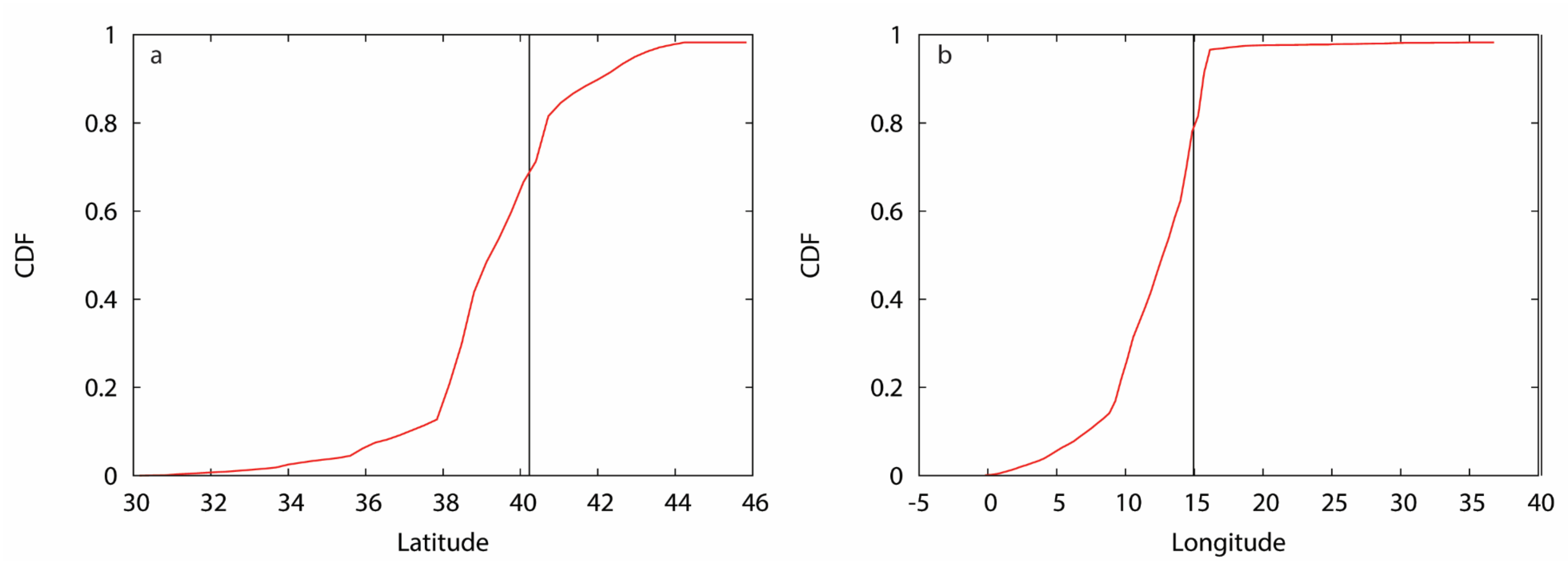

Supplementary Figure S5. Cumulative curves of the final particles' Latitudes (a) and Longitudes (b) for 2013. The vertical bar indicates the position of the release area. A significant amount of particles is observed to move to latitudes north of the release site, where winter SST are lower than in the south Tyrrhenian Sea. This explains why a warming of 2.25°C is needed to reach the threshold of a 0.75 Survival Index despite that winter southern Tyrrhenian SSTs are actually at about 13.5-14°C (i.e., closer to the mortality threshold).

Supplementary Table S1. Occasional loggerhead turtle nests documented in the Western Mediterranean since 2001.

Site	Date of egg laying	Date of 1st emergence	Reference
Vera, ES	27/07/2001	-	Tomas et al. 2002
Palombaggia, FR	-	20/11/2002	Delaugerre and Cesarini, 2004
Geremeas, IT	09/08/2006	-	Bentivegna et al. 2010
Sain Tropez, FR	18/07/2006	-	Senegas et al. 2009
Puzol, ES	11/08/2006	-	Tomas et al. 2008
Premia de Mar, ES	-	27/10/2006	Tomas et al. 2008
Cetraro, IT	-	25/07/2008	Bentivegna et al. 2010
Palermo, IT	-	03/10/2010	Insacco et al 2011
Palermo, IT	11/07/2011	-	Casale et al. 2012
Malgrat de Mar, ES	-	01/10/2011	Carreras et al, 2015
Mondello, IT	15/07/2013		WWF Italy 2013
Scarlino, IT		03/10/2013	Legambiente 2013a
Bonifati, IT	26/06/2013	-	Legambiente 2013b
Bonifati, IT	2013		Legambiente 2013b
Falerna, IT	21/07/2013	-	WWF Calabria 2013
Alicante, ES	30/06/2014	-	Carreras et al, 2015
Tarragona, ES	-	31/10/2014	Carreras et al, 2015
Tarragona, ES	-	30/10/2014	Carreras et al, 2015
Cagliari, IT	11/07/2014	-	de Lucia et al. 2015
Oristano, IT	-	25/09/2014	de Lucia et al. 2015
Sassari, IT	-	02/10/2014	de Lucia et al. 2015
Giannella, IT	-	06/09/2015	WWF Italy 2015
Diamante, IT	-	18/08/2015	Gazzetta del Sud, Calabria 2015
Scauri, IT	29/07/2015	-	SZN unpubblished data
de San Juan de los Terreros, ES	20/07/2015	-	Medio Ambiente, 2015
Torrevieja, ES	31/07/2015	-	Europa Press, 2015

Supplementary Table S1 Reference list

Bentivegna, F. *et al*. Loggerhead turtle (*Caretta caretta*) nests at high latitudes in Italy: a call for vigilance in the Western Mediterranean. *Chelonian Conserv. Bi.* **9,** 283–289 (2010).

Bentivegna, F., Treglia, G. & Hochscheid, S. The first report of a loggerhead turtle *Caretta caretta* nest on the central Tyrrhenian coast (western Mediterranean). *Marine Biodiversity Records* **1,** e14 (2008).

Carreras, C., *et al.* From accidental nesters to potential colonisers, the sequencial colonization of the Mediterranean by the loggerhead sea turtle (*Caretta caretta*). In: Kaska, Y., Sönmez, B., Türkecan, O. & Sezgin, C., editors. *Book of abstracts of 35th Annual Symposium on Sea Turtle Biology and Conservation*. (MACART press, 2015) p. 179.

Casale, P. *et al.* Exceptional sea turtle nest records in 2011 suggest an underestimated nesting potential in Sicily (Italy). *Acta Herpetol.* **7,** 181–188 (2012).

De Lucia, G. A., *et al.* Accidental records of *Caretta caretta* nests in the two main italian island (Sardinia and Sicily) in the last years: can we still talk about occasional nesting areas? In: Kaska, Y., Sönmez, B., Türkecan, O. & Sezgin, C., editors. *Book of abstracts of 35th Annual Symposium on Sea Turtle Biology and Conservation*. (MACART press, 2015) p. 179.

Delaugerre. M & Cesarini, C. Confirmed nesting of the loggerhead turtle in Corsica. *Marine Turtle Newsletter* **104,** 12 (2004).

Europa Press, *Los huevos de tortuga boba de Torrevieja empiezan a eclosionar en la incubadora del Oceanogràfic en Valencia. Newsletter article*. (2015) Available at: http://www.20minutos.es/noticia/2564154/0/huevos-tortuga-boba-torrevieja-empiezan-eclosionar-incubadora-oceanogr-fic-valencia/ (Accessed: 20th November 2015).

Gazzetta del Sud, Calabria, *Nido tartaruga iniziata la schiusa. Newsletter article.* (2015) Available at: http://www.gazzettadelsud.it/news//154955/Nido-tartaruga--iniziata-schiusa.html (Accessed: 20th November 2015).

Insacco, G. *et al.* Sicily 2010 nest season: bad weather and good news. In: Bentivegna, F., Maffucci, F. & Mauriello, V., compilers. *Book of Abstracts: 4th Mediterranean Conference on Marine Turtles*. Naples, Italy, p. 78 (2011).

Legambiente, *Tartarughe marine a Scarlino, dopo le nascite ora monitoraggio e tutela. Greenreport.it. Newsletter article*. (2013a) Available at: http://www.greenreport.it/news/aree-protette-e-biodiversita/tartarughe-marine-a-scarlino-dopo-le-nascite-ora-monitoraggio-e-tutela/ (Accessed: 20th November 2015).

Legambiente, *Trovati 2 nidi di Caretta caretta. Legambiente.* (2013b) Available at: http://www.legambiente.it/contenuti/notizie-dal-territorio/trovati-2-nidi-di-caretta-caretta (Accessed: 20th November 2015).

Medio Ambiente, *Una Tortuga boba desova 80 huevos en una cala de Terreros*. *Newsletter article.* (2015) Available at: http://sevilla.abc.es/andalucia/almeria/20150720/sevi-tortuga-boba-desova-huevos-201507201729.html (Accessed: 20th November 2015).

Sénégas, J.-B., Hochscheid, S., Groul, J.-M., Lagarrigue, B. & Bentivegna, F. Discovery of the northernmost loggerhead sea turtle (*Caretta caretta*) nest. *Marine Biodiversity Records* **2,** e81 (2009).

Tomas, J. *et al*. Is the Spanish coast within the regular nesting range of the Mediterranean loggerhead sea turtle (*Caretta caretta*)? *J. Mar. Biol. Ass. UK* **88,** 1509–1512 (2008).

Tomas, J., Mons, J. L., Martin, J. J., Bellido, J. J. & Castillo, J. J. Study of the first reported nest of loggerhead sea turtle, *Caretta caretta*, in the Spanish Mediterranean coast. *J. Mar. Biol. Ass. UK* **82,** 1005–1007 (2002).

WWF Calabria, *Torna in liberta' la tartaruga " Lucia" mentre il nido di falerna regala le ultime sorprese. la soddisfazione del WWF*. (2013) Available at: http://regionali.wwf.it/client/regionali.aspx?root=8769 (Accessed: 20th November 2015).

WWF Italy, *Tartarughe marine, schiusa a Mondello*. (2013) Available at: http://www.wwf.it/news/notizie/?3460 (Accessed: 20th November 2015).

WWF Italy, *Si schiude un nido di tartaruga marina all'Argentario*. (2015) Available at: http://www.wwf.it/news/notizie/?17960/Si-schiude-un-nido-di-tartaruga-marina-allArgentario (Accessed: 20th November 2015).

Supplementary Table S2. List of model's output, prepared for CMIP5, RCP 4.5 utilized to extract monthly mean SSTs for South Tyrrhenian Sea (12°< Longitude < 16° E, 38° < Latitude < 41° N) since January 1861 to December 2100.

Modeling Center (or Group)	Institute ID	Model Name
Commonwealth Scientific and Industrial Research Organization (CSIRO) and Bureau of Meteorology (BOM), Australia	CSIRO-BOM	ACCESS1.0<br>ACCESS1.3
Beijing Climate Center, China Meteorological Administration	BCC	BCC-CSM1.1<br>BCC-CSM1.1(m)
Canadian Centre for Climate Modelling and Analysis	CCCMA	CanESM2
National Center for Atmospheric Research	NCAR	CCSM4
Community Earth System Model Contributors	NSF-DOE-NCAR	CESM1(BGC)<br>CESM1(CAM5)
Centro Euro-Mediterraneo per I Cambiamenti Climatici	CMCC	CMCC-CM<br>CMCC-CMS
Centre National de Recherches Météorologiques / Centre Européen de Recherche et Formation Avancée en Calcul Scientifique	CNRM-CERFACS	CNRM-CM5
Commonwealth Scientific and Industrial Research Organization in collaboration with Queensland Climate Change Centre of Excellence	CSIRO-QCCCE	CSIRO-Mk3.6.0
EC-EARTH consortium	EC-EARTH	EC-EARTH
The First Institute of Oceanography, SOA, China	FIO	FIO-ESM
NOAA Geophysical Fluid Dynamics Laboratory	NOAA GFDL	GFDL-CM3<br>GFDL-ESM2G<br>GFDL-ESM2M
NASA Goddard Institute for Space Studies	NASA GISS	GISS-E2-H<br>GISS-E2-H-CC<br>GISS-E2-R<br>GISS-E2-R-CC

National Institute of Meteorological Research/Korea Meteorological Administration	NIMR/KMA	HadGEM2-AO
Met Office Hadley Centre (additional HadGEM2-ES realizations contributed by Instituto Nacional de Pesquisas Espaciais)	MOHC<br><br>(additional realizations by INPE)	HadGEM2-CC<br><br>HadGEM2-ES
Institute for Numerical Mathematics	INM	INM-CM4
Institut Pierre-Simon Laplace	IPSL	IPSL-CM5A-LR<br>IPSL-CM5A-MR<br>IPSL-CM5B-LR
Atmosphere and Ocean Research Institute (The University of Tokyo), National Institute for Environmental Studies, and Japan Agency for Marine-Earth Science and Technology	MIROC	MIROC5
Max-Planck-Institut für Meteorologie (Max Planck Institute for Meteorology)	MPI-M	MPI-ESM-MR<br>MPI-ESM-LR
Meteorological Research Institute	MRI	MRI-CGCM3
Norwegian Climate Centre	NCC	NorESM1-M<br>NorESM1-ME
