# Supplementary figures and images for: Seasonal heterogeneity of ocean warming: a mortality sink for ectotherm colonizers
Source: Sci Rep. 2016 Apr 5;6:23983. doi: 10.1038/srep23983 (PMC4820753; doi:10.1038/srep23983)

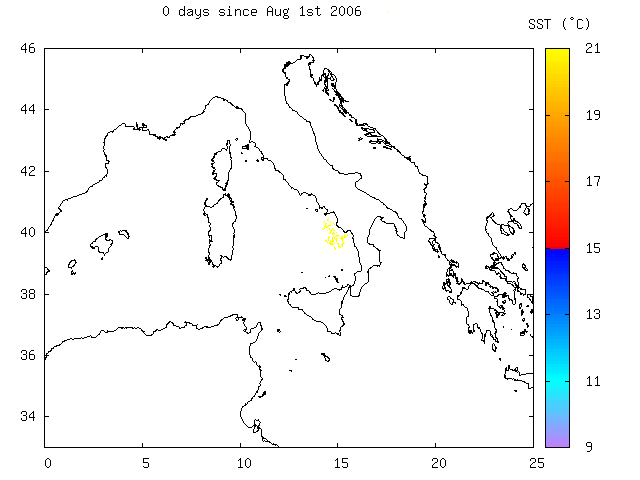

Supplement: Supplementary Figure S4a [file srep23983-s2.gif]

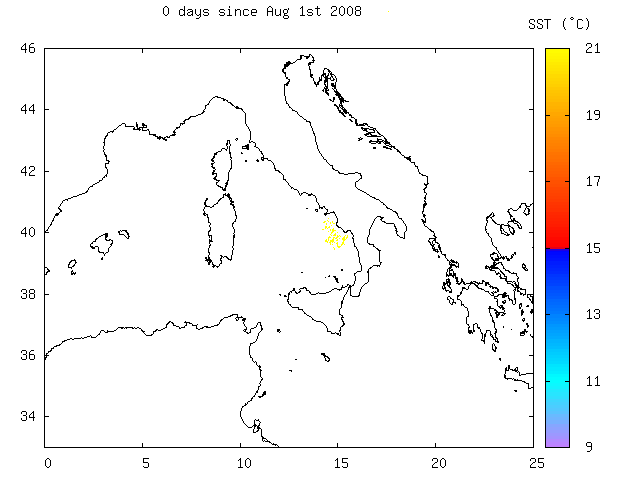

Supplement: Supplementary Figure S4b [file srep23983-s3.gif]

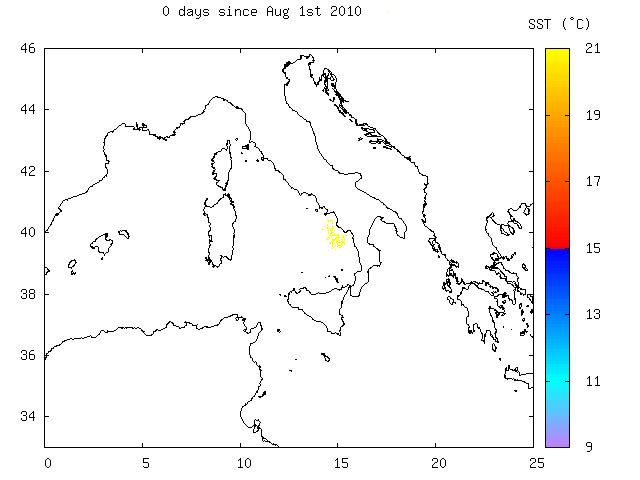

Supplement: Supplementary Figure S4c [file srep23983-s4.gif]
